# Non-invasive nanoscale imaging of protein micro- and nanocrystals for screening crystallization conditions

**DOI:** 10.1107/S1600576724010124

**Published:** 2024-11-22

**Authors:** Krishna Prasad Khakurel, Kei Hosomi, Wataru Inami, Kawata Yoshimasa

**Affiliations:** a Extreme Light Infrastructure (ERIC), Za Radnici 835, 25241Dolni Brezany, Czechia; b Research Institute of Electronics Shizuoka University 3-5-1 Johoku, Chuo-ku Hamamatsu432-8561 Japan; Ecole National Supérieure des Mines, Saint-Etienne, France

**Keywords:** nanoscopy, nanocrystals, protein crystal screening, cathodoluminescence

## Abstract

Crystallography has been the routine technique for studying high-resolution structures of proteins for over five decades. A major bottleneck in structure determination of macromolecules is obtaining crystals of a size and quality suitable for single-crystal X-ray crystallography experiments. Many challenging proteins either fail to grow into crystals or fail to grow into crystals of a size suitable for obtaining high-resolution structures using conventional X-ray crystallography. When it comes to smaller crystals, they can be used either for seeding to get larger crystals or for serial crystallography and electron diffraction for obtaining the structures. For both purposes, a limiting step is to non-invasively image such small crystals of sub-micrometre dimensions and to screen the conditions where such crystals prevail. Here we use cathodoluminescence-based (CL-based) nanoscopy to image protein nanocrystals. We show that crystals of micrometre and submicrometre dimensions can be non-invasively imaged by the CL-based nanoscope. The results presented here demonstrate the feasibility of non-invasive imaging of protein crystals with sub-100 nm resolution.

## Abstract 2

The article presents a non-invasive nanoscale imaging technique that can be used in screening crystallization conditions for protein micro- and nanocrystals.

## 1. Introduction

With the emergence of high-brightness X-ray sources and the development of 3D electron diffraction (3DED), solving the structure of macromolecules from their micrometre and submicrometre crystals has become possible in recent times (Chapman *et al.*, 2011 ▸; Johansson *et al.*, 2017 ▸; Mehrabi *et al.*, 2021 ▸; Khakurel *et al.*, 2019 ▸; Nannenga & Gonen, 2019 ▸). It is challenging to crystallize macromolecules, in particular to obtain crystals of a size and quality suitable for conventional single-crystal X-ray crystallography within a practically reasonable time. However, this challenge persists even with the latest developments. The screening of such tiny crystals prior to data collection and confirmation that the crystallization solution consists of a few crystals have been the major hurdle in making micro/nanocrystallography with bright X-ray and electron sources routine (Lunde *et al.*, 2005 ▸). Any tool in the crystallographer’s toolbox for overcoming this challenge can potentially speed up the study of the structure and dynamics of proteins.

For conventional single-crystal X-ray crystallography, crystals are typically screened using optical microscopes (Nollert, 2003 ▸). The crystal size necessary for such experiments is often of the size of tens of micrometres; hence optical microscopes suffice for imaging such crystals. Further, differentiation of the crystal and aggregates is done using the birefringence property of the protein crystal (Bodenstaff *et al.*, 2002 ▸). With crystals smaller than 10 µm, traditional optical microscopes fail to provide high-resolution images; hence, screening of such crystals poses a challenge (Darmanin *et al.*, 2016 ▸). With micro/nanocrystallography gaining popularity, several other methods such as two-photon UV microscopy (Madden *et al.*, 2011 ▸), second-harmonic generation (Wampler *et al.*, 2008 ▸), and screening crystals with transmission electron microscopy (Stevenson *et al.*, 2014 ▸) and cryo-SEM (scanning electron microscopy) (Beale *et al.*, 2020 ▸) or with environmental SEM (Maruyama *et al.*, 2012 ▸) have emerged. However, none of these methods is non-invasive. Ideally, one needs a non-invasive nanoscope to screen the protein crystals. Here we present the first demonstration of the non-invasive imaging of protein nanocrystals with sub-100 nm resolution, laying the foundation for the non-invasive screening of crystals, in the size range of a few micrometres to less than 1 µm.

We propose using an electron-beam excitation assisted optical microscope (EXA microscope) combined with a scanning electron microscope (Inami *et al.*, 2010 ▸; Kaz *et al.*, 2013 ▸; Nara *et al.*, 2015 ▸) to achieve the non-invasive imaging of the protein crystal. The EXA microscope is a super-resolution microscope, which can provide a spatial resolution of a few tens of nanometres and the capability to image specimens in wet/liquid conditions (Fukuta *et al.*, 2015 ▸; Fukuta *et al.*, 2017 ▸; Nara *et al.*, 2012 ▸). In the microscope, a thin film of cathodoluminescence (CL) material is illuminated by an electron beam, which generates a localized optical probe used for imaging the specimen. A high spatial resolution is achieved by direct electron-beam excitation of the CL thin film and, since the electron beam can be focused in a smaller region than optical light, it can be used as a scanning probe for high-resolution imaging. High-resolution imaging with such a CL-EXA microscope has been demonstrated on several occasions (Kolchiba *et al.*, 2019 ▸; Bischak *et al.*, 2017 ▸). The imaging of wet specimens, nanoparticles and cells with CL-EXA microscopy has attracted the interest of the materials science community in the use of such microscopes. Consequently, efforts to improve the contrast and resolution of the microscope have been pursued in recent times. With the possibility of nano-imaging and by integrating with focused ion beam scanning electron microscopes, the technique can facilitate the non-invasive imaging/screening of protein crystals and further sample preparation for micro/nanocrystallography with X-rays and electrons. In this work, we present the first steps towards achieving this goal.

## 2. Materials and methods

**Figure 1:**
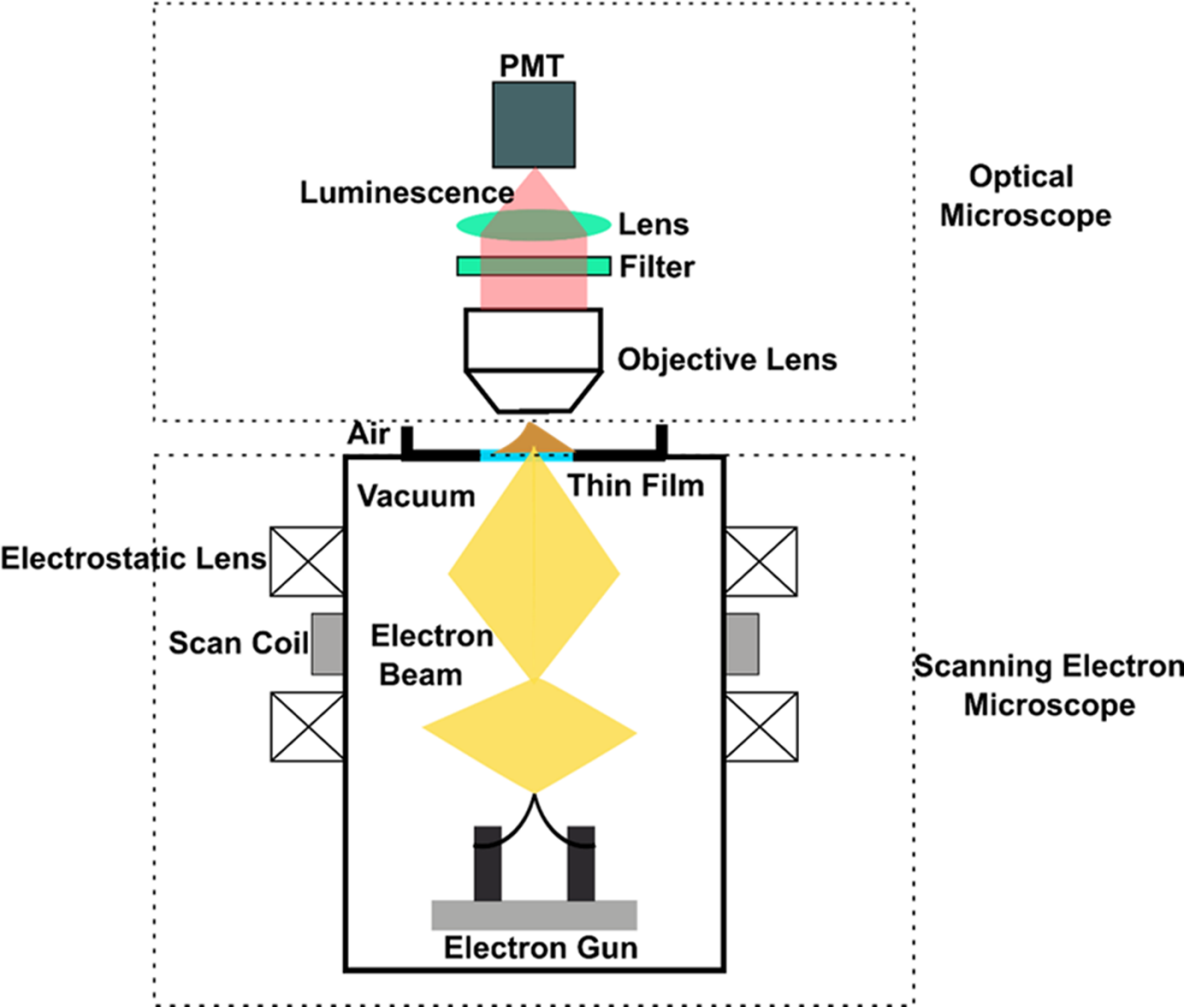
A schematic of the CL-EXA microscope discussed in this article.

The nanoscope that we employ in performing the non-invasive sub-100 nm imaging is based on CL. The central concept behind such a microscope is that a scanning electron beam illuminates a thin film comprising CL material. The local optical probe thus generated acts as the probe for imaging the specimen. Since even low-energy electron beams can be focused down to very small beams, the method can provide a resolution of a few tens of nanometres. A schematic of the CL-EXA microscope is shown in Fig. 1 ▸. The CL-EXA microscope consists of an optical microscope part made from an Olympus BXFM inverted microscope equipped with a CMOS camera (Hamamatsu Photonics C15440-20UP). The scanning electron microscope part of the nanoscope is a Mini-EOC (Apco Co., Japan). The photomultiplier tube (PMT, Hamamatsu Photonics H11461P-11) enables detection of the light transmitted through the sample. A photograph of the CL-EXA microscope and the pipeline used in imaging of the protein crystal is shown in Fig. S1 in the supporting information.

For the current research, we used ZnO as the CL material. ZnO luminescent film was deposited on a silicon nitride (SiN) membrane with a thickness of 50 nm supported on a Si grid. The 50 nm SiN has the mechanical strength to separate ambient air pressure and vacuum. To avoid contamination of the solution containing protein crystals by ZnO thin film, the luminescent films were fabricated onto the vacuum side of the SiN membrane. The deposition of ZnO was conducted by radiofrequency (RF) magnetron sputtering (Sanyu-Electron, SVC-700RF II). A ZnO disc target of 99.99% purity (Kojundo) was sputtered with Ar ions. The working pressure was 1.0 Pa and the RF power was 50 W during sputter deposition. Following the deposition, ZnO/SiN/Si grids were annealed in vacuum at 800°C for 30 min. The heating rate was 5°C min^−1^.

The thickness of the fabricated ZnO thin film was measured to be 100–130 nm with a surface profiler (KLA-Tencor, alpha-step IQ). Surface morphology and luminescent properties were characterized with field emission scanning electron microscopy (FE-SEM, JEOL, JSM-7001F) and a CL optical mirror with a monochromator and a photomultiplier. A typical SEM image and CL spectrum of the ZnO thin film annealed at 800°C for 30 min are shown in Fig. S2. The fabricated ZnO thin film exhibited a sharp luminescent peak at ∼390 nm and a broad emission band around 450–650 nm. The UV emission arises from excitonic emission and the green emission is related to oxygen vacancies. In EXA observations, the whole emission spectrum is used.

**Figure 2:**
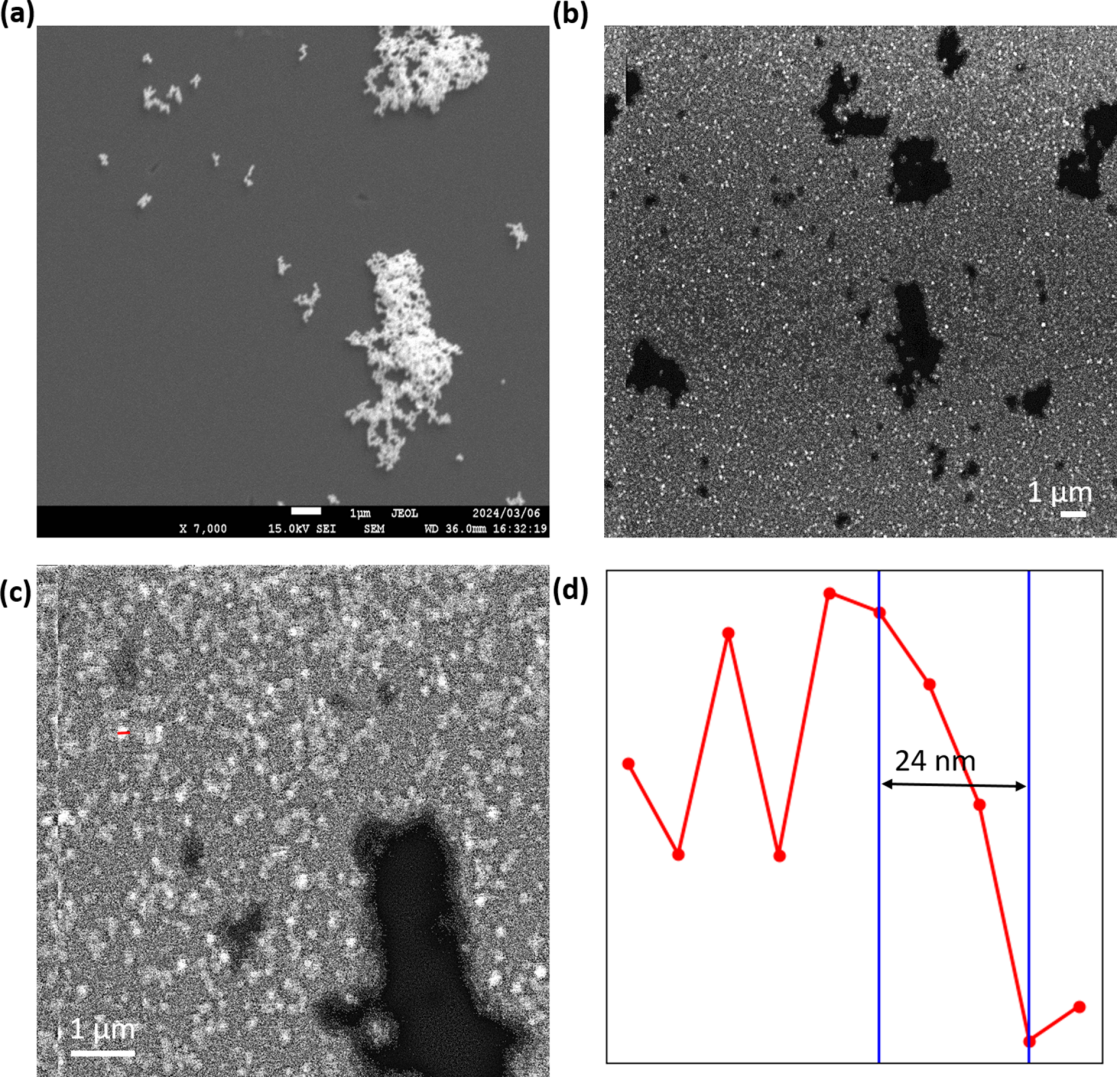
(*a*) Scanning electron micrograph image of the Au NPs. (*b*) The CL-EXA image of the Au NPs at low magnification and (*c*) at high magnification. (*d*) A line plot across the red line shown in (*c*).

An EXA image of Au nanoparticles (NPs) of ∼50 nm in size on the SiN/ZnO membrane is shown in Fig. 2 ▸. The architecture of the SiN/ZnO membrane is illustrated in Fig. S1.

In FE-SEM images, the Au NP aggregates were seen as white objects whereas they were seen in a dark region in EXA images. The CL mode of EXA microscopy detects the light from the luminescent film through the objects. Therefore, the Au NPs were observed as darker in the EXA image. The FE-SEM image of the Au NPs is shown in Fig. 2 ▸(*a*). A low-magnification image obtained from the EXA microscope is shown in Fig. 2 ▸(*b*) and a high-magnification image is shown in Fig. 2 ▸(*c*). The sub-100 nm Au NPs are imaged with high resolution at this magnification. The single-pixel resolution of the EXA microscope at the high-magnification configuration is 8 nm. A line plot along the red line in Fig. 2 ▸(*c*) is shown in Fig. 2 ▸(*d*). From the figure, we can quantitatively determine the edge resolution to be ∼24 nm.

## 3. Monte Carlo simulation

The Monte Carlo simulation estimated the penetration depth of the electron beam in the luminescent film. Monte Carlo simulations of electron trajectories were performed using the *CASINO* v2.51 program (Université de Sherbrooke, Canada). For each computation, 1000 electrons were simulated in an environment consisting of 100 nm of ZnO, 50 nm of SiN and 1000 nm of protein. The composition of protein was set to C_613_H_969_N_193_O_185_S_10_ (Canfield, 1963 ▸), which corresponds to a lysozyme molecule, with density 1.35 g cm^−3^ (Fischer *et al.*, 2004 ▸). All the simulations were performed for an electron energy of 5 kV which was also the energy used for the experiment.

**Figure 3:**
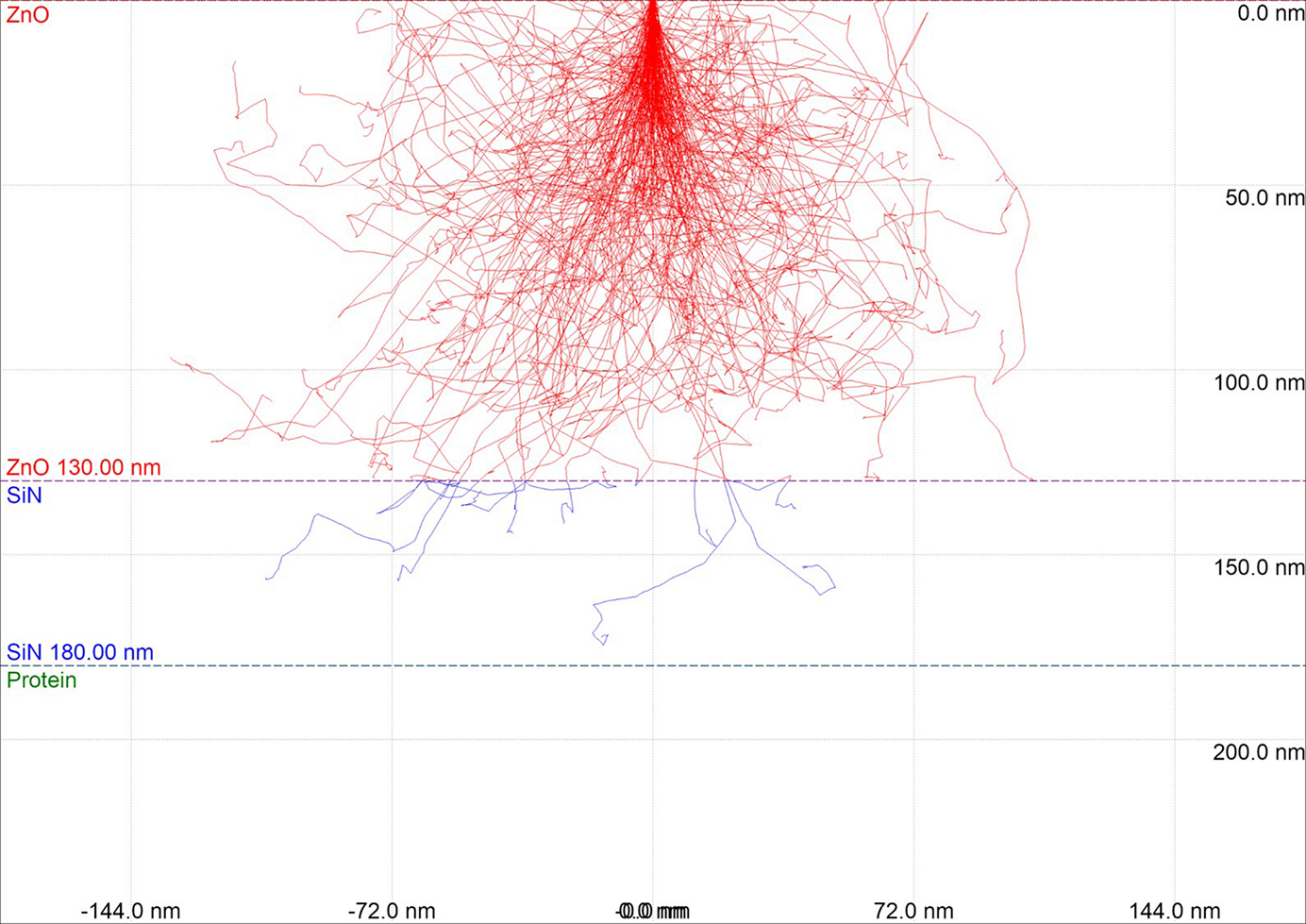
Monte Carlo simulation to estimate the penetration of an electron though the ZnO film to the protein crystal.

Fig. 3 ▸ shows the simulated electron trajectory in ZnO/SiN/protein with ZnO thickness of 100 nm. Although approximately 1% of incident electrons (∼10/1000 electrons) reach the protein in the simulation, almost all electrons are stopped in the ZnO/SiN layer. Thus, non-invasive observation of the sample can be achieved using a ZnO luminescent thin film with a thickness of 100 nm in EXA microscopy. Further, some researchers have previously demonstrated the non-invasive imaging of hydrated cells, which also confirms that protein crystals are undamaged by the electron probe.

## 4. Sample preparation

Lysozyme crystals were fabricated by a batch method. Lysozyme from chicken egg white (Fujifilm Wako Pure Chemical Corporation, Japan) was gently dissolved in a crystallization buffer (1.0 *M* NaCl, 50 m*M* sodium acetate pH 4.6) at a concentration of 100 mg ml^−1^ (Shi *et al.*, 2013 ▸). Subsequently, the solution was stored in a freezer. Lysozyme crystals usually appeared within a few hours.

## 5. Non-invasive imaging of the protein crystal

**Figure 4:**
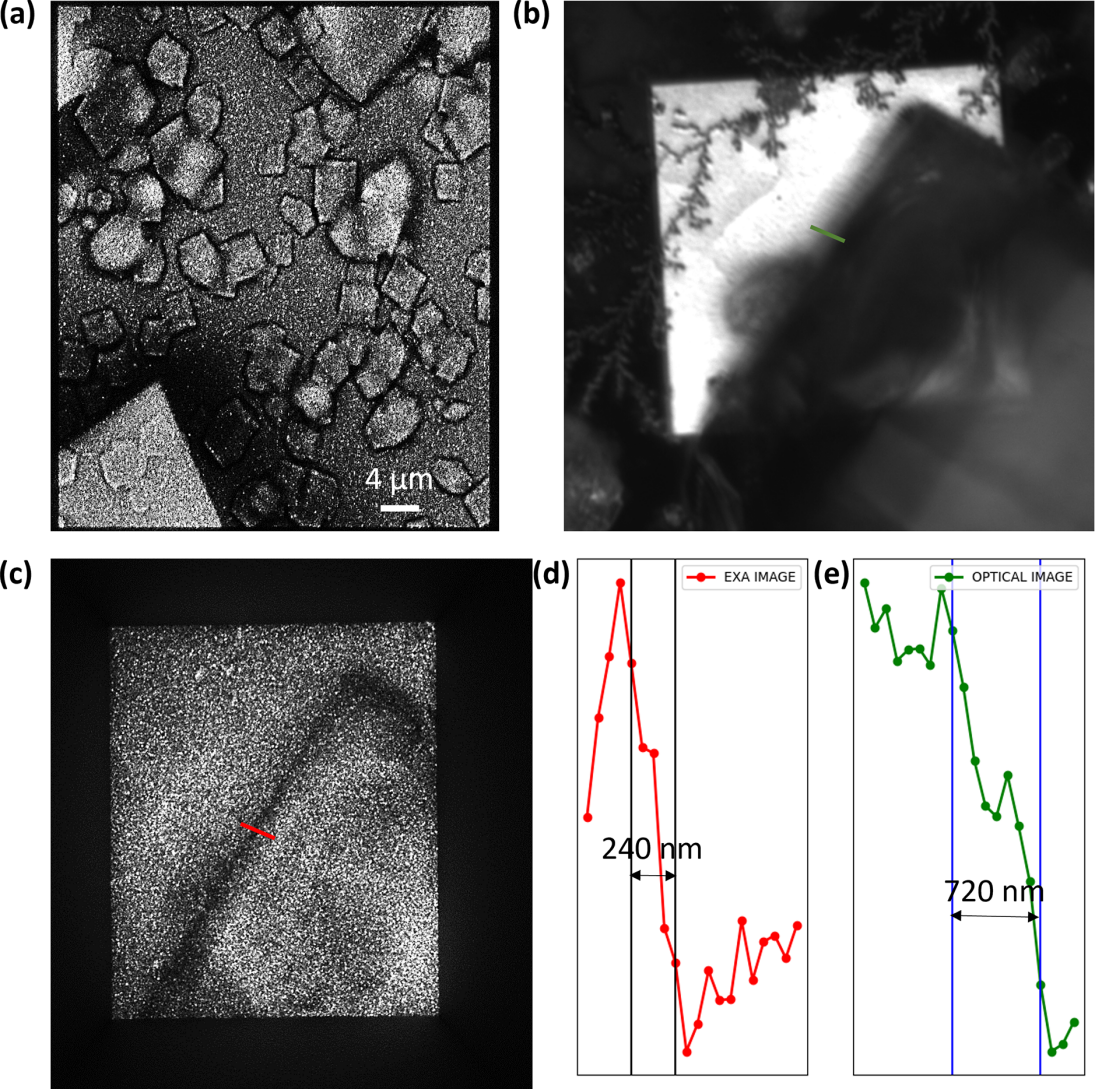
(*a*) CL-EXA images of the protein crystal. (*b*) Optical micrograph of the edge of the protein crystal. (*c*) The CL-EXA image of the edge of the protein crystal. (*d*) A line plot along the red line in (*c*), and (*e*) a line plot along the green line in (*b*).

Micrometre-sized protein crystals were first observed during characterization of the CL microscope. The lysozyme crystals prepared by the method described in the previous section were pipetted to the SiN window. Before observation, we waited for 15–30 min for stabilization of the protein crystals on the surface of SiN. The observation was conducted within a few hours before the solution containing the protein crystals dried out. Fig. 4 ▸(*a*) shows a CL-EXA image. High-quality high-resolution images of protein crystals are obtained from CL-EXA imaging, which would otherwise not be observed in an optical microscope. In order to compare the edge resolution of the optical microscope and the CL-EXA microscope, we took an image of the sharp edge of the protein crystal under a similar optical configuration. The images of the sharp edge of the protein crystal under the optical microscope and the CL-EXA microscope are shown in Figs. 4 ▸(*b*) and 4 ▸(*c*), respectively. Line plots along the edge indicated by the green and red lines in the optical micrograph and the CL-EXA image, respectively, are shown in Figs. 4 ▸(*d*) and 4 ▸(*e*). From the line plots, the edge resolution for the optical micrograph is ∼720 nm and that for the CL-EXA image is ∼240 nm.

We further calculate if any temperature difference is introduced on the sample during the measurements. For the given experimental conditions, the rise in temperature in the ZnO film can be calculated using the equation suggested by Egerton *et al.* (2004 ▸),
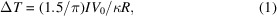
where Δ*T* is the change in temperature, *I* is the probe current, 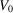 is the accelerating voltage, κ is the thermal conductivity and *R* is the electron range. For the parameters used in the experiment, *I* = 0.5 nA, 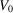 = 5 kV, *R* = 60 nm and κ = 2 W (m K)^−1^ for ZnO taken from Ruoho *et al.* (2015 ▸), the rise in temperature for a stationary probe on the surface of ZnO is ∼10 K. However, for a video rate scanning of the beam the temperature rise is a factor of 10 less, *i.e.* ∼1 K. Therefore, the maximum heating of the sample by the electron-beam irradiation on the ZnO film is ∼1 K, which ensures that the environment of the protein crystal is non-invaded during the imaging.

## 6. Imaging of the protein nanocrystal and correlation with SEM image

**Figure 5:**
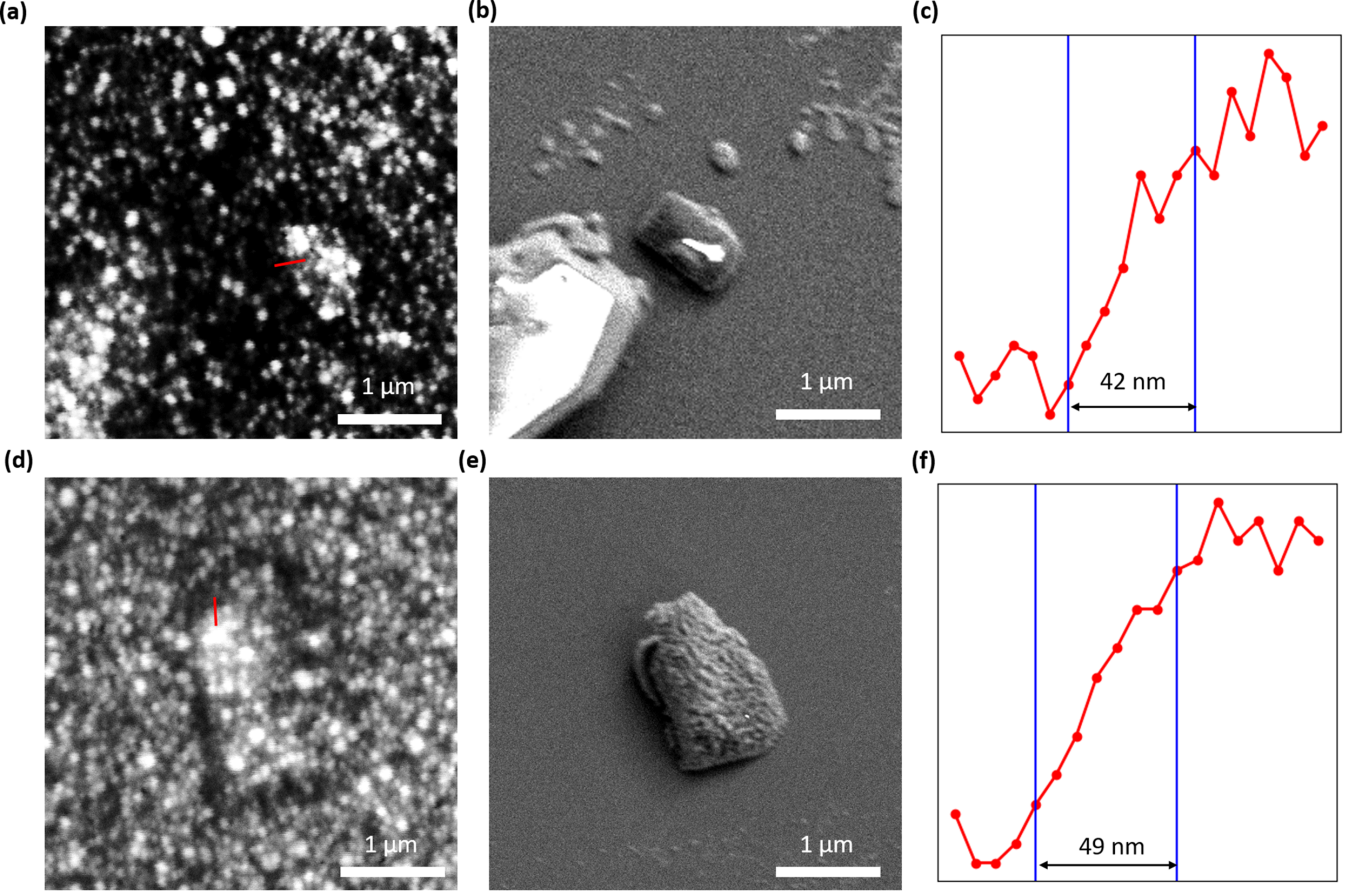
(*a*), (*d*) CL-EXA images of a sub-micrometre protein crystal. (*b*), (*e*) FE-SEM images of the crystal shown in (*a*) and (*d*), respectively. (*c*), (*f*) Line plots along the red lines in (*a*) and (*d*), respectively.

After successfully imaging the protein microcrystal, we have imaged the submicrometre protein crystal using the CL-EXA microscope. The imaging of the protein crystal was done as explained in the previous section. The images in the preceding section were taken in the low-magnification configuration of the microscope. The imaging of the sub-micrometre crystal was done in the high-magnification mode. Figs. 5 ▸(*a*) and 5 ▸(*d*) show images of a protein crystal taken by the CL-EXA microscope. In order to confirm that the objects seen under the CL-EXA microscope were submicrometre crystals of protein, we have also taken FE-SEM images of the same crystal. After EXA imaging, the dish with the crystals was immediately inserted into the FE-SEM chamber, and then the sub-micrometre crystals were observed by FE-SEM at an acceleration voltage of 2 kV. Figs. 5 ▸(*b*) and 5 ▸(*e*) show the FE-SEM images of the crystals corresponding to the CL-EXA images shown in Figs. 5 ▸(*a*) and 5 ▸(*d*), respectively. A line plot along the edges of the crystal as indicated by the red line in Figs. 5 ▸(*a*) and 5 ▸(*b*) is shown in Figs. 5 ▸(*c*) and 5 ▸(*f*), respectively. From the line plot the edge resolutions obtained for the two crystal images from CL-EXA microscopy are 42 and 49 nm, respectively.

## 7. Conclusion and future perspective

We have demonstrated the potential of CL-EXA microscopy in imaging sub-micrometre protein crystals in a non-invasive way. This opens up screening of crystallization conditions in which crystals fail to grow to sizes and of a quality suitable for single-crystal X-ray crystallography. The highly localized excitation can extend the resolution of the microscope to the nanometre scale, thus enabling the high-resolution study of crystallogenesis in a non-invasive way. Our results show that sub-100 nm resolution imaging of a sub-micrometre protein crystal is feasible with the CL-EXA microscope. The microscope, CL thin film and detection system were not particularly optimized for protein crystal imaging. However, with optimization, we believe that high-contrast and high-resolution imaging of protein sub-micrometre crystals can be performed in a routine fashion. Furthermore, use of optical fibers optimized to collect polarized light and their use in analogy to traditional polarized optical microscopy can enhance the contrast of the image, aiding differentiation of the crystal from the aggregates. With the advances in machine learning in image processing, the contrast of the CL-EXA images can also be improved by adaptation of those tools.

Correlative light electron microscopy has also been a widely used tool in the cryo-electron microscopy community. Our results of non-invasive nano-imaging of a biological specimen can contribute to such correlative light electron microscopy efforts with CL-based microscopes. Interest in the use of such correlative microscopes in 3DED of small crystals and crystals buried under the crystallization medium has been reported previously. We believe the results presented here and the prospect that CL spectra can be acquired across a wide spectral range to enable identification of specific proteins will facilitate such efforts. We also believe that, with further advancement and optimization of the CL-EXA microscope, it could be introduced into the routine pipeline for screening protein crystals.

## Supplementary Material

Supplementary figures. DOI: 10.1107/S1600576724010124/nb5386sup1.pdf
