# Supplementary material for: Non-invasive nanoscale imaging of protein micro- and nanocrystals for screening crystallization conditions
Source: J Appl Crystallogr. 2024 Nov 22;57(Pt 6):1907–12. doi: 10.1107/S1600576724010124 (PMC11611282; doi:10.1107/S1600576724010124)
Supplement: Supplementary file 1 [file j-57-01907-sup1.pdf]

JOURNAL OF
APPLIED
CRYSTALLOGRAPHY

Volume 57 (2024)

Supporting information for article:

Non-invasive nanoscale imaging of protein micro- and nano-crystals for screening crystallization conditions

Krishna Prasad Khakurel, Kei Hosomi, Wataru Inami and Kawata Yoshimasa

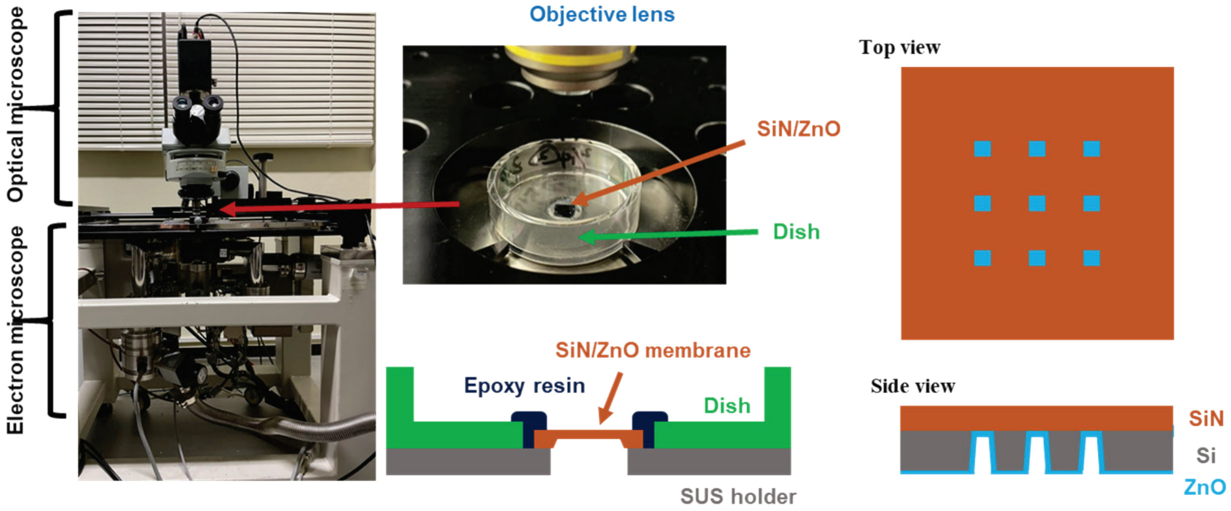

Figure S1 The photograph of the EXA-CL microscope and the pipeline of protocol of imaging of the micro/nanocrystals of proteins.

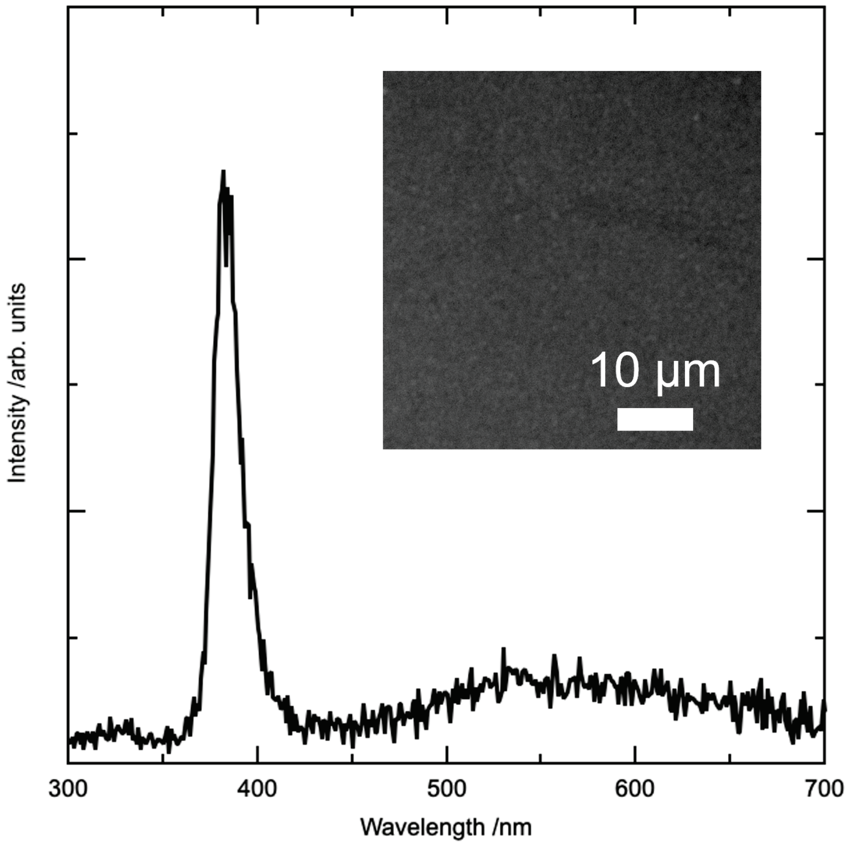

Figure S2 A typical cathodoluminescence spectra of ZnO. The inset shows the SEM image of the ZnO thin film.
